# Abc3-Mediated Efflux of an Endogenous Digoxin-like Steroidal Glycoside by *Magnaporthe oryzae* Is Necessary for Host Invasion during Blast Disease

**DOI:** 10.1371/journal.ppat.1002888

**Published:** 2012-08-23

**Authors:** Rajesh N. Patkar, Yang Kui Xue, Guanghou Shui, Markus R. Wenk, Naweed I. Naqvi

**Affiliations:** 1 Temasek Life Sciences Laboratory and Department of Biological Sciences, National University of Singapore, Singapore, Singapore; 2 Department of Biochemistry, Yong Loo Lin School of Medicine, National University of Singapore, Singapore, Singapore; 3 School of Biological Sciences, Nanyang Technological University, Singapore, Singapore; Purdue University, United States of America

## Abstract

*Magnaporthe oryzae*, which causes the devastating rice-blast disease, invades its host plants via a specialized infection structure called the appressorium. Previously, we showed that the ATP-Binding Cassette 3 transporter is necessary for appressorial function (host penetration) in *M. oryzae*. However, thus far, the molecular basis underlying impaired appressorial function in the *abc3*Δ remains elusive. We hypothesized that the *abc3*Δ appressoria accumulate excessive amounts of specific efflux substrate(s) of the Abc3 transporter in *M. oryzae*. We devised an innovative yeast-based strategy and identified Abc3 Transporter efflux Substrate (ATS) to be a digoxin-like endogenous steroidal glycoside that accumulates to inhibitory levels in *M. oryzae abc3*Δ appressoria. Exogenous ATS altered cell wall biogenesis and viability in wild-type *Schizosaccharomyces pombe*, but not in *S. pombe* expressing *M. oryzae* Abc3. We show that ATS associates with the Translation Elongation factor Tef2 in *M. oryzae*, and propose that ATS regulates ion homeostasis during pathogenesis. Excessive ATS accumulation, either intracellularly due to impaired efflux in the *abc3*Δ or when added exogenously to the wild type, renders *M. oryzae* nonpathogenic. Furthermore, we demonstrate that the host penetration defects in the *abc3*Δ are due to aberrant F-actin dynamics as a result of altered Tef2 function and/or ion homeostasis defects caused by excess accumulation of ATS therein. Rather surprisingly, excessive exogenous ATS or digoxin elicited the hypersensitive response in rice, even in the absence of the blast fungus. Lastly, reduced disease symptoms in the inoculated host plants in the presence of excessive digoxin suggest a potential use for such related steroidal glycosides in controlling rice-blast disease.

## Author Summary

*Magnaporthe oryzae*, the causal fungus of the devastating blast disease in rice, invades its host via specialized infection structures called appressoria. Previously, we showed that ATP-Binding Cassette 3 (Abc3) transporter is indispensable for appresssorial function of host penetration in *M. oryzae*. However, the cause of inviable appressoria and impaired host entry in the *abc3*Δ remained unclear. ABC transporters are known to efflux xenobiotic or toxic molecules to the cell exterior. Therefore, we hypothesized that the loss of Abc3 pump leads to excessive accumulation of its physiological substrate to likely inhibitory levels resulting in appressorial dysfunction. We devised an innovative yeast-based strategy to successfully purify the Abc3 Transporter Substrate (ATS). We show that ATS is a digoxin-like endogenous steroidal glycoside primarily involved in modulating ion homeostasis and host colonization in *M. oryzae*. Furthermore, we identified Translational Elongation Factor 2 (Tef2) as the target for ATS, and find a mechanistic link between ATS, ion homeostasis, Tef2 function, and F-actin dynamics during *M. oryzae* pathogenesis. We uncover a unique ability of ATS to induce the hypersensitive response and consequently disease resistance in host plants. Lastly, digoxin-like steroidal glycosides promise to be novel antifungal agents to combat the destructive blast disease in crop plants.

## Introduction

ATP-binding cassette (ABC) transporters are able to couple the binding and hydrolysis of ATP to efflux a variety of toxic molecules such as antifungal, antibacterial, or anticancer agents [1], [2]. Often over-expression of ABC transporters confers multidrug resistance (MDR) and hence is believed to be an adaptive albeit opportunistic mechanism to protect cells from various toxic entities [3], [4]. However, apart from MDR, it is possible that each ABC transporter serves a distinct physiological function and effluxes a specific natural substrate including an endogenous metabolite. For example, P-glycoprotein (P-gp) at the apical membrane in nephrons is a well-characterized transporter of the steroidal glycoside digoxin [5], [6] and likely effluxes other member(s) of the endogenous Digoxin-like Immunoreactive Factor (DLIF) family. Since identification of the specific physiological efflux substrate is a daunting task that remains largely unaccomplished, MDR remains the only assigned function for most ABC transporters [2], [7].

Several bacterial ABC transporters are required to secrete toxins and antimicrobial agents [8]. Similarly, fungal pathogens likely utilize ABC transporters to keep host-derived antimicrobial substances at bay, and in addition efflux compounds involved in virulence [9]. Phytopathogens synthesize low molecular weight compounds (secondary metabolites), that are bioactive and in some instances required for virulence [10], [11] but not for growth *per se*. Such metabolites may be secreted out or effluxed by virulence-associated pumps. For example, *Cochliobolus carbonum* produces a maize-specific virulence factor called HC-toxin, a cyclic tetrapeptide inhibitor [12], which is hypothesized to be effluxed by the ToxA and ToxB transporters [13]. ABC-transporters BcatrB from *Botrytis cenerea* and GpABC1 in *Gibberella pulicaris* are similarly required for resistance towards respective host-derived phytoalexins resveratrol and rishitin [14], [15].

*M. oryzae*, an ascomycete and the causal agent of rice blast disease, undergoes pathogenic differentiation upon contact with the host, wherein the asexual spore/conidium develops into a germ tube, which elongates and develops into a specialized infection structure called the appressorium [16], [17]. The appressoria generate enormous turgor pressure and mechanically breach the cuticle (appressorial function) to enter the host plants. It has been proposed that *M. oryzae* deploys an efficient and effective strategy, wherein the fungus secretes a large array of specific virulence factors (elicitors and/or effectors) into the host, to prepare it for the invasion and to cope with the stress therein [18], [19]. Interestingly, host plants have evolved a highly-efficient strategy to recognize specific effectors or elicitors, in order to activate the defense response (Hypersensitive Response or HR) to control the pathogen spread [20], [21], [22]. A rapid efflux of K^+^ and an influx of Ca^+2^ and H^+^ ions mark the first phase of HR induction in plants. The second phase of the HR includes elevation of reactive oxygen species (ROS), increase in levels of phenolics, and induction of pathogenesis related (PR) genes [23], [24], [25].

*M. oryzae* genome encodes about 50 ABC transporters [9], of which four have been characterized thus far for their role(s) in fungal pathogenesis. While Abc1, Abc3, and Abc4 are required for effective virulence, Abc2 is dispensable for pathogenesis in *M. oryzae* [7], [26], [27], [28]. However, none of these ABC transporters has been assigned any physiological function or is known to efflux a specific substrate in *M. oryzae*. Previously, we have shown that Abc3, which localizes predominantly to the plasma membrane in the appressoria, is essential for the host-penetration step during pathogenesis in *M. oryzae* [27]. It has been proposed that loss of pathogenicity in the *abc3*Δ mutant is likely due to excess appressorial accumulation of the physiological efflux substrate of the Abc3 pump [27].

In the present work, we identify an endogenous metabolite, ATS, as the specific efflux substrate of the Abc3 transporter in *M. oryzae*. We show that Abc3 activity is essential for efflux of ATS. We characterize the likely functions of ATS that strike a mechanistic link between ion homeostasis, Tef2-function and modulation of the actin cytoskeleton in *M. oryzae*. Finally, we propose that ATS serves as an important component that alters the host response and outcome of the *M. oryzae*-Rice interaction during initiation of the blast disease.

## Results/Discussion

### *M. oryzae abc3*Δ mutant accumulates a cytotoxic molecule

**Figure 1:**
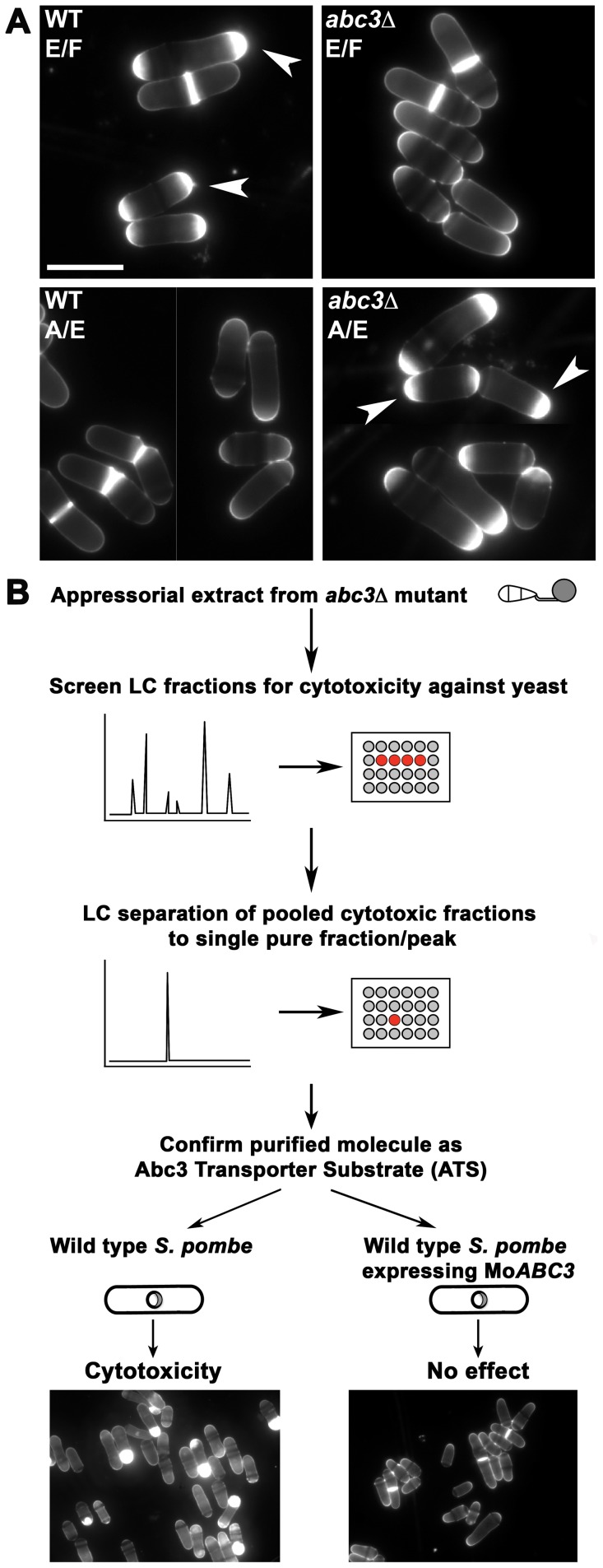
Isolation of ATS from *abc3*Δ appressoria in *M. oryzae*. (**A**) Wild-type *S. pombe* cells were treated with extracellular fluid (E/F) or appressorial extract (A/E) from the wild-type or *abc3*Δ *M. oryzae* strain for 6 h and stained with calcofluor white (CFW). Arrowheads indicate aberrant deposition of septal/cell wall material at the cell tip(s). Bars = 10 µm. (**B**) Schematic representation of the *S. pombe* cell-based assay used to guide the purification of ATS and to confirm ATS as an efflux substrate of the Abc3 transporter. Mo*ABC3* refers to *M. oryzae ABC3*.

Previously, we showed that loss of Abc3 transporter-function leads to impaired host penetration in *M. oryzae* [27]. Based on the predominant localization of Abc3 to the plasma membrane of the appressorium, it was hypothesized that accumulation of the endogenous efflux substrate therein was responsible for cell death in the *abc3*Δ mutant [27]. A suitable tool was necessary to guide the purification of such a cytotoxic moiety, presumably the efflux target of Abc3. Interestingly, appressorial extracts from *abc3*Δ led to cell enlargement, aberrant and excessive septal/cell wall deposition at cell ends (Figure 1A), and consequent loss of viability in wild type fission yeast. However, treatment with total extracts from wild-type appressoria did not lead to such defects or cell death in yeast. To check whether the cytotoxic activity was present in the appressorial exterior, we tested the effect of the extracellular fluid surrounding the wild-type or *abc3*Δ appressoria on yeast. Importantly, the cell wall biogenesis defects were evident only in yeast treated with extracellular fluid from the wild type and not that of the *abc3*Δ strain (Figure 1A). We proceeded to utilize such cytotoxicity-based assay as a tool to guide purification (Figure 1B) of the endogenous molecule (ATS) from appressorial extracts of the *abc3*Δ mutant. We reckoned that the maximum amount of ATS would accumulate by 24 hpi since Abc3-GFP translocates thereafter from the appressorial plasma membrane to the vacuoles [27]. Molecular size-based fractionation of the *abc3*Δ appressorial extract was carried out where a few of the resulting subfractions contained the cytotoxic activity against the wild type *S. pombe*. Such cytotoxic fractions were further resolved by size-based separation, and each individual purified fraction tested in the aforementioned yeast cell based assay. Fractions containing significant cytotoxic activity were pooled and purified further using reverse phase FPLC. Finally, the cytotoxic activity against the wild-type *S. pombe* was narrowed down to a fraction that showed a single prominent peak upon UV detection at 196 nm/220 nm. To verify if the purified cytotoxic molecule was a specific efflux target of Abc3, we expressed *M. oryzae* Abc3 transporter in the wild-type *S. pombe* (wild type *S. pombe* expressing Mo*ABC3*) cells. Most importantly, *S. pombe* strain expressing Abc3 did not show any substantial defects or abnormalities in the presence of the purified cytotoxic moeity (Figure 1B), which was thus designated as ATS. Such Abc3-expressing *S. pombe* cells showed normal cell size with medial septa and cytokinesis even in the presence of ATS (or total *abc3*Δ appressorial extract) when compared to the control cells. It is worth noting that expression of a single *M. oryzae* protein, Abc3, negated the inhibitory effect of ATS on *S. pombe*, thus helping in isolation of the target substrate. We conclude that *abc3*Δ appressoria accumulate ATS, which is normally present outside wild-type infection structures, in excess and that ATS is most likely a specific efflux substrate of the Abc3 transporter in *M. oryzae*.

### ATS is a steroidal glycoside that shares structural and functional properties with digoxin

**Figure 2:**
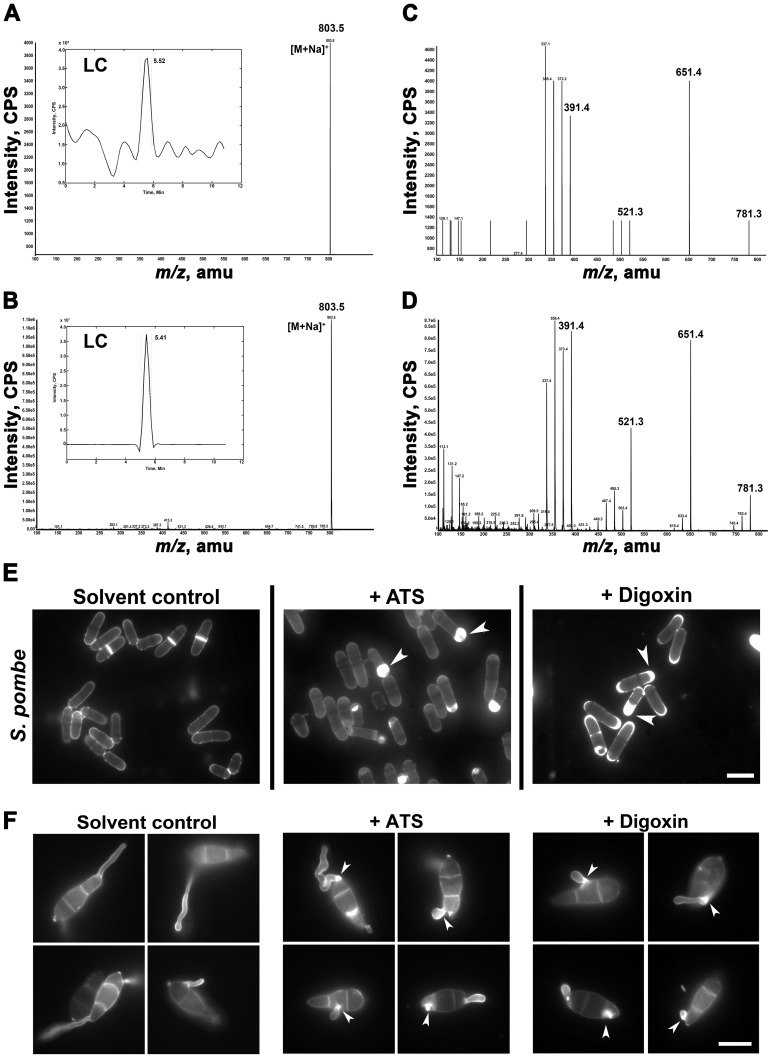
ATS shares structural and functional properties with digoxin. Molecular mass of ATS (**A**) or digoxin (**B**) identified by APCI method. Molecular masses shown are sodium adducts of ATS or digoxin (both, *m/z* 780). Insets depict the predominant peaks of ATS or digoxin with their respective retention times. (**C**) and (**D**) Tandem mass spectra of ATS and digoxin, respectively. The ionization products characteristic of the steroidal nucleus (*m/z* 390), mono- and bi-sugar (*m/z* 520 and 650, respectively) molecules are highlighted. (**E**) Wild-type *S. pombe* cells were treated with residual solvent, ATS, or digoxin for 6 h and stained with CFW. Arrowheads show aberrant septal/cell wall biogenesis. Bars = 5 µm. (**F**) Conidia from wild-type *M. oryzae* were germinated on agarose in the presence of residual solvent, ATS, or digoxin and stained with CFW after 4 h. Excess cell wall deposits are indicated with arrowheads. Bar = 10 µm.

The purified ATS showed a retention time of 5.52 min on the RP-HPLC column (Figure 2A inset). Mass spectrometric analysis by Atmospheric Pressure Chemical Ionization (APCI/MS) of purified ATS showed a major peak with the *m/z* 780 (Na^+^ adduct = *m*/*z* 803.5) (Figure 2A). Reference and compound library searches indicated that digoxin, which is a steroidal glycoside from the foxglove plant, shows a similar molecular mass as ATS. Standard digoxin showed retention time of 5.41 min on RP-HPLC column and *m/z* 780 (Na^+^-adduct = *m*/*z* 803.5) (Figure 2B inset). Tandem mass spectra of digoxin resulted in major fragments with *m/z* 651.4, 521.3, and 391.4, which are successive breakdown products of digitoxose molecules (Figure 2D). Similarly, ATS, upon tandem MS, resulted in major fragments with molecular masses of *m/z* 651.4, 521.3, and 391.4 (Figure 2C) strongly indicating a structural similarity between ATS and digoxin. ELISA tests confirmed the immuno-reactivity of monoclonal anti-digoxin antibodies towards ATS (Figure S1A in Text S1) and helped estimate the ATS concentration in the extracellular fluid and appressorial extracts of the wild type or *abc3*Δ mutant. The concentration of ATS was estimated to be 6 and 7 ng in the total extracellular fluid surrounding the wild type appressoria and *abc3*Δ mutant appressorial extract, respectively, from 1×10^8^ conidia (Figure S1C in Text S1). The *abc3*Δ extracellular fluid and wild type appressorial extract did not show any detectable reactivity towards anti-digoxin antibodies (Asterisks, Figure S1C in Text S1). Similarly, ATS concentration was found to be 0.2 µg/ml in the FPLC-purified fraction (Figure S1B in Text S1); and approximately 500 ng per 2.5×10^8^ appressoria. Although extremely low in concentration, the amount of ATS extracted per unit biomass was considerably higher in mature appressoria than in vegetative hyphae. Based on the above analyses, *M. oryzae* ATS was thus considered to be an endogenous digoxin-like steroidal glycoside.

To test if digoxin shared the cytotoxic property of ATS, we studied the growth kinetics of wild-type or Abc3-expressing *S. pombe* cells treated with ATS or digoxin. Wild-type or Abc3-expressing *S. pombe* cells were grown in the presence of different concentrations of ATS or digoxin, and the cell density measured every hour over a 24 h period. The cell density of the wild-type *S. pombe* culture showed substantially reduced growth rate after 4 h of ATS or digoxin treatment, whereas the cells treated with residual solvent showed growth kinetics similar to that of the untreated control. Growth curves indicated that incubation for 6 h was sufficient to observe the inhibitory effect of ATS or digoxin on *S. pombe*. While the wild-type *S. pombe* showed a dose-dependent inhibitory activity of ATS (Figure S2A in Text S1), the Abc3-expressing cells did not show any considerable difference in the growth kinetics in the presence of ATS (Figure S2B in Text S1). Similarly, digoxin showed inhibitory effect in a dose-dependent manner towards wild-type yeast (Figure S2C in Text S1), but not on the Abc3-expressing yeast cells (Figure S2D in Text S1). The minimum concentration of digoxin required to completely inhibit growth in the wild-type *S. pombe* cells was found to be 125 µM. Digoxigenin (an aglycone derivative of digoxin) or Ouabain (a related steroidal glycoside) showed similar potency in inhibiting yeast growth when compared to digoxin (Figure S3A and Figure S3B in Text S1). Hereafter, we refer to the cytotoxic effect of ATS or digoxin as inhibitory activity.

Next, qualitative bioassays were performed using wild-type *S. pombe* cells and ATS or digoxin using the same assay conditions described above and observed after 6 h. Indeed, the wild-type *S. pombe* cells treated with digoxin showed similar defects in septal/cell wall deposition and cell size (Figure 2E) as elicited upon ATS treatment, indicating that inhibition of cell growth is likely due to (hitherto unknown) inhibitory activity of digoxin similar to that of ATS. Additionally, ATS- or digoxin-treated wild-type *Saccharomyces cerevisiae* (BY4741) and *Candida albicans* (SC5314) showed enlarged cells with excess and aberrant septal/cell wall deposits predominantly at the bud neck (Figure S2E in Text S1). The induction of hyphal growth in *C. albicans* was not completely inhibited by ATS or digoxin; however, hyphal elongation was considerably restricted with similar defects in septum/cell wall biogenesis (Figure S2E in Text S1). Similarly, on a non-inductive surface, ATS- or digoxin-treated conidia of the wild-type *M. oryzae* resulted in short and curved germ tubes with excessive cell wall deposits at the point of emergence (Figure 2F).

We tested if ATS shows the characteristic digoxin-like activity on heart function. Interestingly, ATS-treated zebrafish larvae showed considerably reduced heart rate similar to that ascribed to digoxin treatment. While zebrafish embryos treated with approximately 415 nM (100 ng/300 µl fish water) of ATS showed no obvious effect on the development of the larvae, ATS reduced the heart rates substantially in a dose-dependent manner when compared to residual solvent-treated embryos until 48 hours post fertilization (hpf). Heart rates were found to be 94.8±5.0, 84.6±0.2, and 88.0±6.9 beats/min in the control, ATS-, or digoxin-treated larvae, respectively, at 26 hpf (*P* = 0.0108; Figure S4 in Text S1; Video S1 and Video S2). Based on similarities between ATS and digoxin, and the most likely role of ATS during appressorial function, an analogy could be drawn between the heart and the fungal appressorium. Both these structures require hydrodynamic turgor for their respective functions; however, proper contraction of the heart is brought about by highly regulated ion fluxes across the membranes of the cardiomyocytes. This suggests a similar function for ATS in regulating ion homeostasis during appressorium formation and/or function at the onset of host entry.

Endogenous DLIFs were discovered in part as a consequence of their cross-reactivity to anti-digoxin antibodies. Various studies suggest that such endogenous DLIFs and Ouabain-like factors (OLFs) not only are similar in structure but also share function with digoxin and ouabain, respectively. Interestingly, although analysis by techniques such as mass spectrometry suggested that OLF was similar to ouabain, later studies using exciton-coupled circular dichroism showed that OLF is structurally distinct from ouabain [29], [30]. Thus, although ATS and digoxin show immuno-reactivity with anti-digoxin antibodies and share certain functional similarities, we do not rule out subtle structural and physical differences such as those predicted in DLIFs or OLFs when compared to digoxin. Our repeated attempts at NMR-based analysis of ATS structure have been largely unsuccessful given the extremely low concentration of ATS produced by the fungal appressoria. Similar difficulties have been encountered in characterization of DLIFs from mammalian tissues [29]. Although the anabolic pathway for endogenous cardiac glycoside (CG) biosynthesis is not fully clear, Qazzaz et al. [6] suggest three critical steps in the transformation of the last known precursor Pregnenolone or Progesterone into a CG. Identification of such critical enzymes in digoxin biosynthesis, although elusive, would certainly enable further characterization of the endogenous CGs (and ATS) through overexpression and/or mutagenesis approach.

Thus, ATS shares the structural properties and an inhibitory activity with digoxin. We uncovered a hitherto uncharacterized dose-dependent and broad spectrum inhibitory activity of digoxin. Taken together, we conclude that ATS is a DLIF or steroidal glycoside that shows structural and functional relatedness to digoxin. Furthermore, we concur that excess ATS or digoxin perturbs the cell wall biogenesis machinery in yeast and in *M. oryzae*.

### Inhibitory activity of ATS is associated with Tef2-function in *M. oryzae*

**Figure 3:**
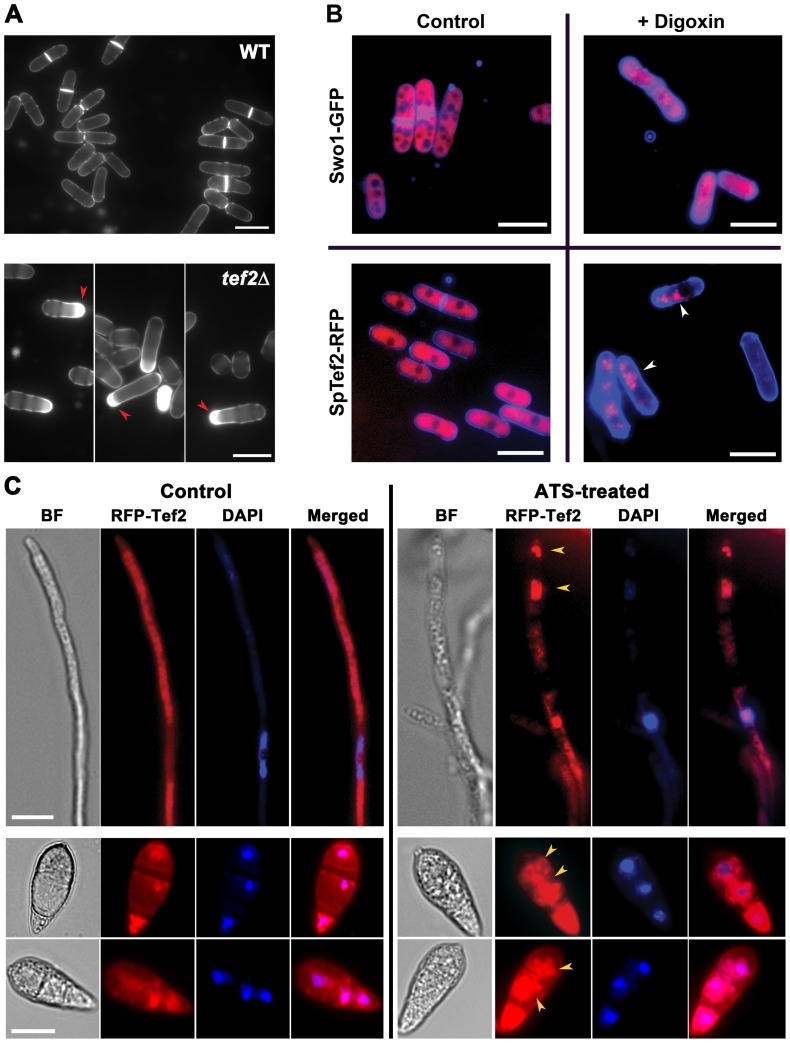
ATS associates with Tef2 in *S. pombe* and *M. oryzae*. (**A**) Loss of SpTef2-function simulates ATS effect in *S. pombe*. Cell wall staining of the wild-type or *tef2*Δ *S. pombe* cells using CFW. Red arrowheads depict defective septal/cell wall deposition. Scale bar equals 10 micron. (**B**) Effect of digoxin on subcellular localization of SpTef2-RFP or Swo1-GFP in *S. pombe* cells. The strains expressing the indicated fusion proteins were stained with CFW and analysed by epifluorescence microscopy. Arrowheads show distinct aggregates of SpTef2-RFP. Bar = 10 µm. (**C**) Effect of ATS on localization of RFP-Tef2 in *M. oryzae* vegetative hyphae (upper panels; Scale Bar = 5 µm) and conidia (middle and lower panels; Bar represents 10 µm) co-stained with DAPI to aid visualization of nuclei. Arrowheads denote aberrant perinuclear aggregates and/or patches of RFP-Tef2. BF, Bright Field.

To identify the downstream target(s) and to understand the mechanism underlying the intracellular function of ATS, we performed a pull-down assay using monoclonal anti-digoxin antibodies that specifically recognised ATS too. Total protein extracts from the wild-type *M. oryzae* strain was incubated with or without ATS, and standard immunoprecipitation was carried out with monoclonal anti-digoxin antibodies. A 55 kDa polypeptide was detected specifically in the ATS-treated pull-down fraction, but was absent in the untreated control (Figure S5A in Text S1). Mass spectrometric analysis identified this protein as the Translation Elongation Factor 2 (GenPept: XP_361098.1; Tef2, alias eEF1A2) ortholog from *M. oryzae* (Figure S5B in Text S1; *P*<0.05). Absence of Tef2 in the control (- ATS; untreated) pull-down ruled out a possibility that the co-immunoprecipitation was due to sheer abundance (if any) of Tef2. *TEF2* was found to be an essential gene in *M. oryzae* since a gene-deletion mutant for *TEF2* could not be obtained even after repeated attempts. In all such attempts, only the transformants with random integration of the gene-deletion construct could be recovered. Notably, the *tef2*Δ *S. pombe* strain showed septal/cell wall deposition defects similar to those observed in the wild-type yeast treated with ATS or digoxin (Figure 3A). Furthermore, an SpTef2-RFP fusion protein localized to the cytoplasm in control cells (Figure 3B), however, it appeared predominantly in the form of distinct aggregates in the digoxin-treated cells that showed the characteristic cell wall/septal abnormalities (Figure 3B, lower panels). To ascertain whether the aggregation was due to the activity of digoxin or shrinkage in the cytoplasm, we studied the localization of a known cytoplasmic protein Swo1 (Hsp90) in digoxin-treated cells. Uniform cytoplasmic distribution of Swo1-GFP (Hsp90-GFP), upon treatment with digoxin, confirmed the aggregation of SpTef2-RFP and supported a specific association with the steroidal glycoside (Figure 3B, upper panels). Similarly, RFP-Tef2 fusion protein was predominantly cytosolic in vegetative hyphae and conidia in *M. oryzae*. In addition to being cytosolic, RFP-Tef2 localized to nuclear and perinuclear regions in *M. oryzae* conidia (Figure 3C). However, distinct and highly intense cytosolic and perinuclear aggregates of RFP-Tef2 were evident in ATS-treated mycelia and conidia in wild-type *M. oryzae* (Figure 3C). We infer that ATS physically associates with Tef2 in *M. oryzae*, and that the inhibitory activity of ATS or digoxin is likely coupled with alteration in the function of Tef2.

### Functional relationship between ATS, ion homeostasis, Tef2-function, and the F-actin cytoskeleton

**Figure 4:**
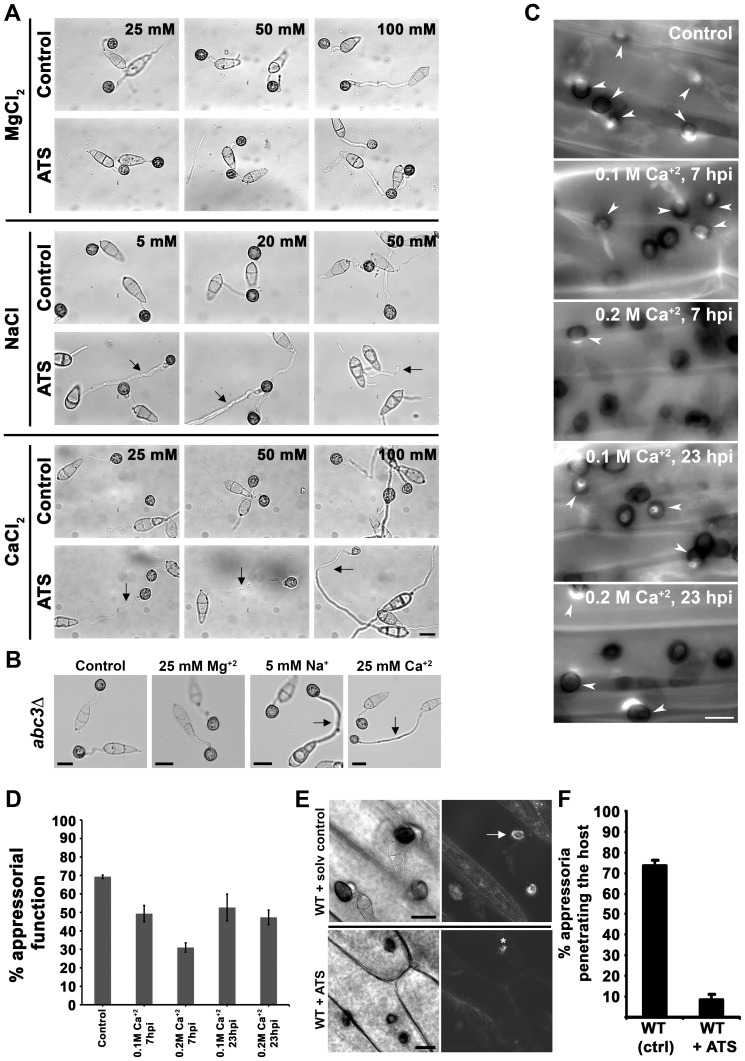
ATS plays a role in ion homeostasis during pathogenesis in *M. oryzae*. (**A**) ATS increases sensitivity of wild-type *M. oryzae* towards specific cations. Excess or permissive concentrations of Ca^+2^, Na^+^, or Mg^+2^ ion were added to the germinating wild-type conidia in the presence or absence of ATS. Arrows show delayed appressorial development (longer germ tubes) in the presence of ATS, which was otherwise seen only in the presence of excess concentration of the ions under control condition. Bar = 10 µm. (**B**) Sensitivity of the *abc3*Δ towards permissive concentratios of indicated cations. Arrows indicate delayed response in terms of longer germ tubes. Bars = 10 µm. (**C**) Effect of excess Ca^+2^ on appressorial function/host penetration efficiency in *M. oryzae*. Penetration efficiency was evaluated at 28 hpi by staining callose deposits with Aniline Blue. Arrowheads depict appressoria successful in host penetration. Bar = 10 µm. (**D**) Penetration efficiency of the appressoria was calculated as % appressorial function at 28 hpi. Data represent mean ± SEM from 3 individual experiments (n = 100 each per replicate). (**E**) Rice leaf sheaths were inoculated with wild-type *M. oryzae* in the presence of residual solvent or ATS for 24 h, and stained with aniline blue (right panels) for induced callose deposits (arrow) underneath the sites of host penetration (appressorial function). Asterisk shows occasional callose deposition. Bars = 10 µm. (**F**) Quantification of appressorial function at 30 hpi. The data represents mean ± SEM from 3 individual assays.

Digoxin is known to inhibit the Na^+^/K^+^ ATPase pump in the membranes of the cardiac myocytes leading to an increase of intracellular Na^+^, followed by Ca^+2^ ions [31]. To indirectly test whether ATS shows a similar mode of action as a patho-physiological function, we studied appressorial development in wild-type *M. oryzae* in the presence of excess ATS and Na^+^ or Ca^+2^ ions. To rule out a possibility of non-specific response to any cation, we also tested the effect of Mg^+2^ ions in parallel. Wild-type *M. oryzae* developed appressoria with normal germ tube length and incubation time (6 to 8 h) in the presence of permissive concentration of Na^+^ (5 and 20 mM NaCl), Ca^+2^ (25 and 50 mM CaCl_2_), or Mg^+2^ ions (25 and 50 mM MgCl_2_) as in case of untreated control (Figure 4A). On the other hand, wild-type *M. oryzae* showed delayed appressorial development (16 to 20 h) in the presence of excess Na^+^ (50 mM NaCl), Ca^+2^ (100 mM CaCl_2_), or Mg^+2^ (100 mM MgCl_2_) (Figure 4A). Interestingly, ATS addition delayed appressorial development even in the presence of permissive concentration of Na^+^ or Ca^+2^ (Figure 4A, middle and lower panels) in wild type *M. oryzae*. Notably, normal appressorial development was evident in the presence of ATS and permissive concentration of Mg^+2^ indicating a specific response towards Na^+^ and Ca^+2^ ions during appressorial development. Importantly, the *abc3*Δ mutant showed similar sensitivity specifically towards Na^+^ and Ca^+2^ during appressorial development (Figure 4B). These results suggest that ATS, under physiological conditions, may be involved in regulating intracellular levels of Na^+^ and Ca^+2^ ions during hydrodynamic conditions prevalent in appressorial development in *M. oryzae*. Ingold had hypothesized that glucose and ions were essential for generating the turgor pressure sufficient for forcible discharge of ascospores in *Sordaria fimicola* [32]. Indeed, an elegant study has shown that mannitol is not enough to generate the turgor pressure sufficient for discharge of the eight ascospores in *Gibberella zeae*, but the K^+^ and Cl^−^ ions present in the ascus fluid are necessary to generate the required force [33]. Since Ca^+2^-mediated signaling plays a crucial role and is studied extensively in most eukaryotes, we decided to focus more on the effect of Ca^+2^ ions for further analysis of ion homeostasis in *M. oryzae*. In order to study the importance of ion homeostasis during pathogenesis, we tested the effect of excess Ca^+2^ on appressorial function in *M. oryzae* through quantification of callose deposits as an indicator of host penetration. Aniline blue-stained callose deposits were evident underneath 70% of the untreated wild-type appressoria at 30 hpi. Whereas, only 50 or 30% of the appressoria were capable of entering the host in the presence of 0.1 or 0.2 M Ca^+2^, respectively, added at 7 hpi (Figure 4C and 4D; *P* = 0.001). Similarly, penetration efficiency of the appressoria was reduced to 50 or 45% when 0.1 or 0.2 M Ca^+2^, respectively, was added at 23 hpi (Figure 4C and 4D; *P* = 0.0016). Further, we studied the effect of excess ATS on host penetration by wild type *M. oryzae*. While, 73.3±2.7% of the wild-type untreated appressoria showed normal host-penetration efficiency at 30–36 hpi, only 8.6±2.6% of the ATS-treated wild-type appressoria were able to induce callose deposition in the host tissue (Figure 4E and 4F, *P* = 0.00003). Microscopic observation after 30 hpi revealed that only 5–10% of the ATS-treated wild-type appressoria could develop invasive hyphae as opposed to 60% in the untreated control. Our previous studies showed that the *abc3*Δ appressoria were significantly defective in penetrating the host tissue likely due to intracellular accumulation of ATS [27]. These results indicate a significantly reduced appressorial function in the presence of excess endogenous or exogenous ATS. Altogether, we conclude that Ca^+2^ flux plays an important role during appressorial development as well as function; and deduce that endogenous ATS likely serves a physiological role in regulating such ion homeostasis in *M. oryzae*.

**Figure 5:**
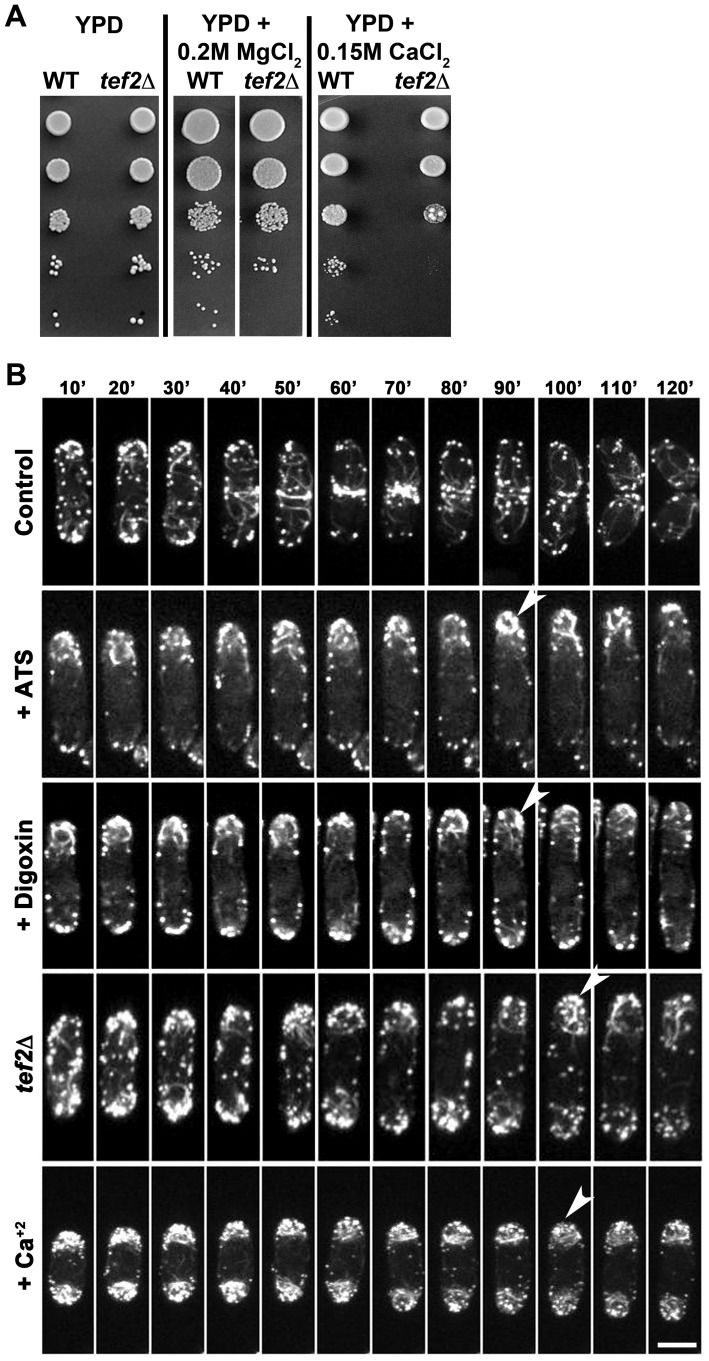
SpTef2 function and the F-actin cytoskeleton in *S. pombe*. (**A**) Sensitivity of *tef2*Δ *S. pombe* cells towards Ca^+2^ in the growth medium. Serial dilutions of the wild-type or *tef2*Δ cells were inoculated under indicated growth conditions. (**B**) Morphology and dynamics of GFP-labelled F-actin cytoskeleton in wild-type *S. pombe* treated with ATS, digoxin or Ca^+2^. The *tef2*Δ strain was analyzed in parallel. Arrowheads show excess accumulation of F-actin patches and/or short, spooling cables at the cell end(s). The maximum projection images shown here represent the compressed z-stack sections. Bar equals 10 µm.

Tef2 or eEF1A2 is one of the two isoforms of the translation elongation factor eEF1A (Tef1 or eEF1A1 and Tef2 or eEF1A2). While eEF1A1 is ubiquitously expressed, eEF1A2 is found mainly in heart, brain, and skeletal muscle [34], [35], [36] indicating that these isoforms may have differential functions other than their canonical role(s) in translation elongation. To test if there is any association between Tef2 and ion homeostasis, we analyzed the sensitivity of the *tef2*Δ *S. pombe* strain towards Ca^+2^ or Mg^+2^ in the growth medium. While both the wild-type and *tef2*Δ cells grew normally on YPD or YPD supplemented with 0.2 M MgCl_2_, the *tef2*Δ strain showed significant sensitivity towards and growth inhibition in 0.15 M CaCl_2_ (Figure 5A). This suggests a possible non-canonical function for SpTef2 in ion homeostasis in *S. pombe*. Indeed, Kaur and Ruben have shown that EF-1α directly interacts with calmodulin CaM that is involved in calcium signaling in protozoan parasite *Trypanosoma brucei* [37].

**Figure 6:**
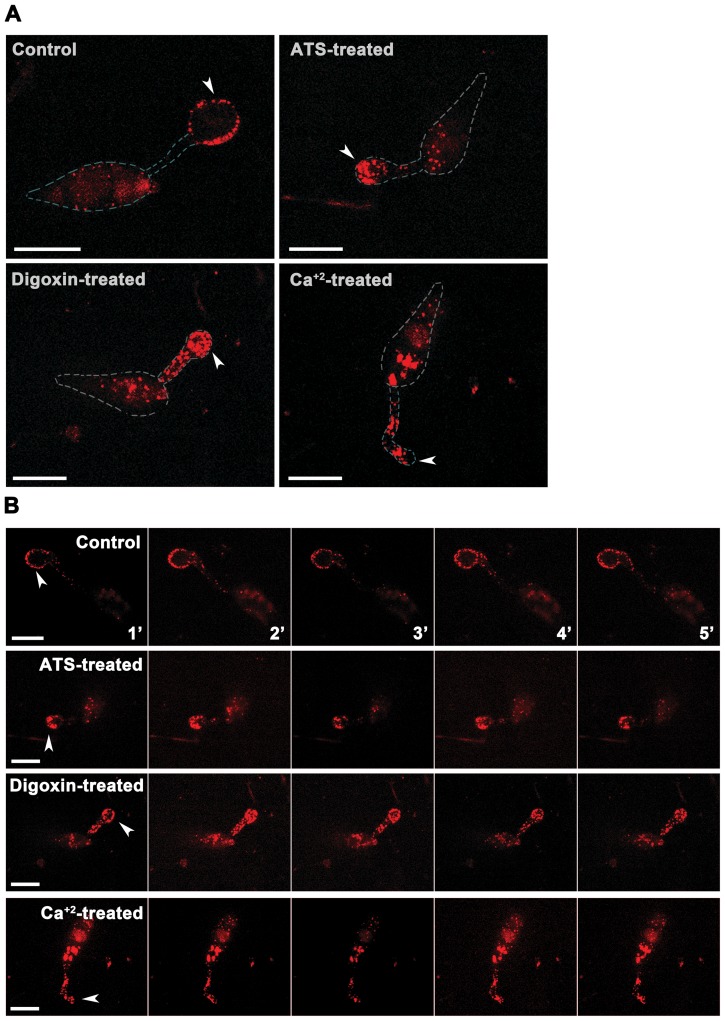
Exogenous ATS or digoxin alters the F-actin cytoskeleton in *M. oryzae*. Morphology (**A**) and dynamics (**B**) of the F-actin patches in wild type *M. oryzae* expressing Abp1-RFP and treated with ATS, digoxin, or 0.1 M CaCl_2_. Arrowheads depict developing appressoria. Bars = 10 µm.

In *Dictyostelium*, Tef2 binds to F-actin and enhances actin bundling, suggesting that it has other cellular functions including actin remodeling [38], [39]. It has been estimated that out of total Tef2 present, >60% could be associated with the actin cytoskeleton in *Dictyostelium* [40]. Similarly, Tef2s from a number of species have been shown to bind actin filaments and/or microtubules both *in vitro* and *in vivo* [41]. Furthermore, calcium signalling induced self-incompatibility in *Papaver rhoeas* leading to inhibition of pollen tube growth is also associated with altered actin cytoskeleton [42]. Therefore, we studied the F-actin organization and dynamics in the *tef2*Δ and wild-type *S. pombe* cells treated with ATS, digoxin, or excess calcium. Normal actomyosin rings were assembled, which was followed by cytokinesis in control wild type *S. pombe* (Figure 5B; Video S3). However, majority of the *tef2*Δ or ATS/digoxin-treated wild-type *S. pombe* cells showed deferred assembly and constriction of actomyosin rings, leading to delayed or failed cytokinesis resulting in elongated and enlarged cells. Interestingly, cells that were unsuccessful in cytokinesis continued growing further with short and spooling F-actin cables at the cell end(s) (Figure 5B; Video S4; Video S5 and Video S6). In addition, the F-actin patches in such ATS- or digoxin-treated cells accumulated predominantly at the cell end(s) and were occasionally dynamic albeit only along the cell periphery (Figure 5B). Notably, wild-type *S. pombe* cells grown in the presence of 0.15 M CaCl_2_ showed similar F-actin morphology and dynamics as observed in ATS- or digoxin-treated cells except that the cell size was considerably smaller when compared to the untreated control cells (Figure 5B; Video S7). Similar excess accumulation of F-actin patches and cables at the cell tip(s) was evident in wild-type *S. pombe* cells treated with either ATS or digoxin, and stained with Alexa Fluor 488 Phalloidin (Figure S6 in Text S1). Further, we studied F-actin organization (Figure 6A) and dynamics (Figure 6B) in *M. oryzae* expressing Actin-Binding Protein 1 (Abp1)-RFP fusion protein. Under control condition, Abp1-RFP-marked cortical actin patches were enriched at the germ tube tips and along the periphery of the developing appressorium in wild type *M. oryzae* (Figure 6A). However, ATS, digoxin, or Ca^+2^- treated wild type *M. oryzae* showed aberrant aggregates of actin patches accumulated in the appressoria or distributed randomly along the germ tubes (Figure 6A). Substantially aberrant localization/morphology and dynamics of F-actin cytoskeleton in treated *M. oryzae* and *S. pombe* cells suggests that ATS (or digoxin) associates with more than one factor and supports a mechanistic link between ion homeostasis, Tef2, and F-actin cytoskeletal organization in yeast/fungi.

Altogether, we propose that under physiological conditions ATS likely regulates ion homeostasis during appressorial function in *M. oryzae*. We further show that excess ATS or digoxin possibly alters the actin cytoskeleton, leading to septal/cell wall biogenesis defect in yeast and *M. oryzae*, and that aberrant Tef2-function and/or calcium signaling is associated with such cytoskeletal remodelling activity.

### Excess ATS or digoxin induces host cell death and reduces rice blast disease symptoms

**Figure 7:**
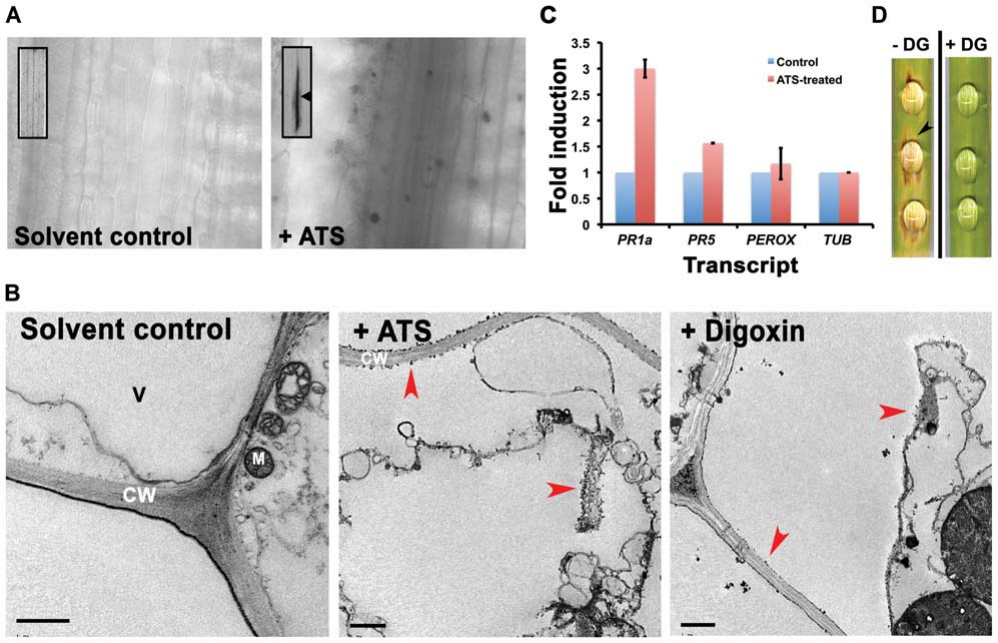
Excess ATS or digoxin induces cell death in the host plants and reduces blast disease severity. (**A**) Barley leaf explants were treated with residual solvent or ATS for 72 h, stained with trypan blue and observed using bright field optics. Arrowhead and arrows show visible (inset) and localized cell death, respectively, in the inoculation zone. (**B**) Transmission electron micrographs of residual solvent-, ATS- or digoxin-treated rice leaf explant stained with CeCl_3_ after 48 h of treatment. Arrowheads depict cerium perhydroxide granules and/or plasmolysis after ATS or digoxin treatment for 48 h. CW, cell wall; M, mitochondrion; and V, vacuole. Bars = 1 µm. (**C**) Transcript levels of Pathogenesis Related genes tested by real-time qRT-PCR in rice after 24 h of treatment. Data represent mean ± SEM of two independent experiments with three replicates each. Perox, peroxidase; Tub, tubulin. (**D**) Detached barley leaf pieces were inoculated with wild-type conidia in the absence or presence of 200 µM digoxin (DG). The disease symptoms were evaluated at 6 dpi. Arrowhead denotes disease lesion. The data represents observations from 3 independent experiments.

Based on Abc3 localization [27] and the presence of ATS activity in the extracellular fluid surrounding the wild-type appressoria, we infer that ATS would be normally effluxed by the Abc3 transporter during pathogenic development in *M. oryzae*. In addition, we hypothesize that a steroidal glycoside such as ATS may have an ability to alter the function of ion transporters in the host membranes (similar to digoxin-based block of Na^+^/K^+^ pump in cardiac myocytes) or target Tef2-like cellular proteins leading to induced host response. Therefore, we tested if ATS has any effect on the host plants. Plant immunity or Hypersensitive Response (HR) is manifested by many ways including localised cell death, oxidative burst, and upregulation of pathogenesis related (PR) proteins. Cell viability tests using trypan blue staining showed visible localised cell death in rice leaf tissue treated with ATS for 48 to 72 h when compared to untreated control samples (Figure 7A). ATS- or digoxin-treated rice leaf sheath was stained with cerium chloride (CeCl_3_) and observed under Transmission Electron Microscope (TEM) to study the oxidative burst, precisely elevated levels of H_2_O_2_. Cerium perhydroxide granules, formed by the reaction of cerium ions with H_2_O_2_, were observed predominantly in the cell wall and cell membrane of the ATS- or digoxin-treated rice leaf tissues. Moreover, the host cells also showed plasmolysis upon treatment with digoxin or ATS when compared to the control leaf tissue, which did not show any plasmolysis or cerium perhydroxide enrichment at the cell wall or plasma membrane (Figure 7B). Furthermore, we analyzed transcript levels of pathogenesis-related genes in the host, including *PR1a*, *PR5*, and peroxidase by quantitative real-time RT-PCR (qRT-PCR) using ATS-treated rice leaves. ATS induced *PR1a*, *PR5*, and peroxidase transcript levels in rice by 3±0.1 , 1.5±0.0, and 1.18±0.3 fold, respectively, at 24 hpi (Figure 7C; *P* = 0.00006). Thus, these observations indicate that ATS or digoxin is capable of inducing an HR in rice.

Since, HR is indicative of host resistance against pathogen attack, we performed a detached-leaf infection assay to test if higher concentrations of digoxin could control blast disease in barley. Barley leaf explants were inoculated with conidia from wild-type *M. oryzae* (ca 100) with or without digoxin, and the disease reaction was scored for lesions at 7 dpi. The inoculated leaf pieces without digoxin started developing blast disease symptoms on day 3. However, equivalent number of conidia in the presence of 200 µM digoxin failed to elicit any considerable disease symptoms. The severity of disease in the untreated control leaves was substantially higher when compared to the digoxin-treated leaves (Figure 7D). Overall, these findings show that ATS or digoxin induces host response in rice and barley, and excess digoxin reduces blast disease in barley.

**Figure 8:**
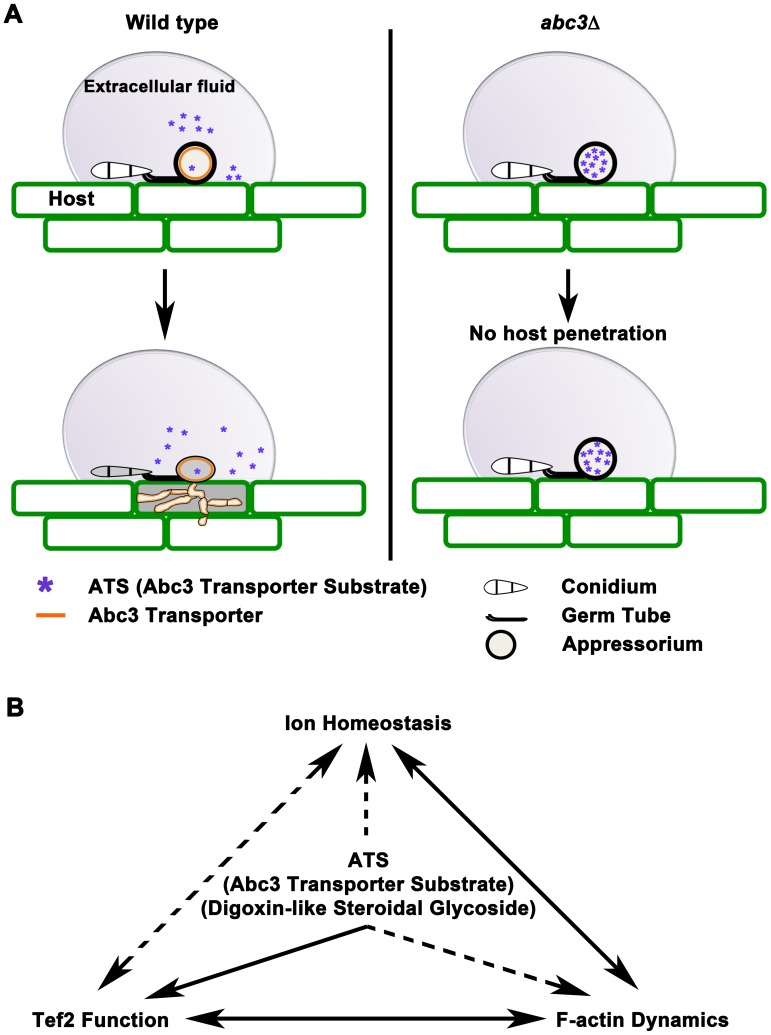
Working model for the role of ATS in *M. oryzae* pathogenesis. (**A**) Schematic representation of accumulation of ATS, in the wild type or *abc3*Δ appressoria, affecting host entry. (**B**) The figure illustrates a proposed crosstalk/mechanistic link between ATS accumulation and ion homeostasis, Tef2-function, and F-actin dynamics during *M. oryzae* pathogenesis.

In conclusion, we identified ATS as a natural efflux substrate of the Abc3 transporter, and showed that excess accumulation of ATS blocks host entry in *M. oryzae* (Figure 8A). Based on the *abc3*Δ defects in the appressoria [27], we infer that ATS may not function directly in pathways that mediate melanin deposition and/or turgor generation. At physiological levels, ATS may contribute to general fitness and integrity of *M. oryzae* by regulating ion homeostasis during appressorial function. However, at excess concentrations ATS represents a previously undescribed activity of fungal metabolites that deregulates cell-wall biogenesis through modulated ion homeostasis and the non-canonical function of Tef2 affecting actin cytoskeleton (Figure 8B) in yeast and fungi.

Mac1, which catalyzes cAMP production from ATP; and *CPKA* that encodes a catalytic subunit of cAMP-dependent Protein Kinase A play important roles in appressorial function [43], [44] and Ca^+2^ mediated signaling. However, exogenous cAMP did not rescue appressorial defects in *abc3*Δ *M. oryzae* suggesting that ATS may function independent of the cAMP signaling pathway. Nonetheless, it would be interesting to analyze whether CpkA is involved in regulating ATS levels and/or ion homeostasis in appressoria. Recently, a serine-rich protein, Defense Suppressor 1 or Des1, has been identified as an important repressor of basal host defense in *M. oryzae* [45]. Des1 is necessary for regulating oxidative stress response in *M. oryzae*, but unlike the Abc3 transporter, is directly involved in the secretion of extracellular peroxidases and laccases. In addition to ROS detoxification, Des1 is also necessary for maintaining the intracellular levels of Ferrous ions [45]. It remains to be seen whether Des1 is required for regulating ion homeostasis, and whether ATS levels stay unperturbed in the *des1*Δ appressoria in *M. oryzae*.

Abc3-GFP does localize to penetration and invasive hyphae, but it remains to be seen whether ATS is effluxed into the host tissue during such *in-planta* growth of *M. oryzae*. Our repeated attempts at detecting ATS *in planta*, via immunoEM using the anti-digoxin antisera, have been largely unsuccessful. However, excess ATS (or digoxin) not only induces host immunity in rice, but also reduces blast disease symptoms, thus suggesting a potential use of these related steroidal glycosides in controlling blast disease in host plants. It is also worth noting that excess ATS or digoxin, specifically blocks appressorial function (without affecting the overall growth *per se*) in *M. oryzae*, and shows potential to define a new paradigm for design of antimicrobial agents. It would be fascinating to analyse (1) how widespread the Abc3/ATS system is in other fungal pathogens and (2) whether ATS-based induction of HR could potentially be useful in restricting other pathogens too. Further analyses are imperative to identify the minimal chemical structure of ATS that is essential to propose better-suited antifungal agent(s). Lastly, the *S. pombe* cell-based assay used to identify ATS in the present study promises to be a powerful tool to screen novel drugs and their targets and to establish ABC transporter-substrate relationships.

## Materials and Methods

### Yeast and fungal cultures

Wild-type *M. oryzae oryzae* strain Guy11 (*mat1-2*) was a kind gift from Didier Tharreau (CIRAD, France). *M. oryzae* strains were propagated on Prune-agar (PA) medium or complete medium (CM) as described [46]. Genetic transformation of *M. oryzae* was carried out as described earlier [27].

Appressorium formation was tested on inductive surfaces (rice leaf sheath, barley leaf explants, or hydrophobic glass coverslips) in the presence of ATS (∼5 ng) or with different (permissive or non-permissive) concentrations of MgCl_2_, NaCl or CaCl_2_. The appressoria formed were observed at 24 hpi using bright field microscopy.

The *S. pombe* strains used in this study are listed in Table S2 (in Text S1). Cells were cultured and maintained using standard techniques [47]. *S. pombe* cells used for the cytotoxicity assays were grown in the YES medium [47].

One-step PCR-based gene deletion using the *URA4* ^+^ marker was performed according to Bahler et al. [48] using an 80-bp flanking sequence homologies. Deletion of the Sp*TEF2* ORF (SPCC794.09c) was performed in the *S. pombe* wild type MBY104. Stable transformants from minimal media (MM) minus uracil were tested and Sp*TEF2*-deletion was confirmed by colony PCR.

Plasmid pFGL547 was created to generate a *S. pombe* strain that expressed a SpTef2-RFP fusion protein from its genomic locus. The complete 1.3 kb orf of the Sp*TEF2* gene was amplified by PCR using primers listed in Table S1 (in Text S1). The 1.3 kb *Sal*I-Sp*TEF2*-*BamH*I fragment was directionally cloned upstream and fused in frame with the RFP gene in the pJK210 RFP plasmid. Plasmid pFGL547 was linearized with *Nhe*I and introduced by electroporation into MBY104 to obtain the Sp*TEF2-RFP* strain. Strains were confirmed by requisite colony PCR and nucleotide sequencing. Genetic crosses were performed by mixing appropriate strains of opposite mating type on YPD plates, followed by selection of recombinant strains by tetrad dissection using an MSM micromanipulator (Singer Instruments, UK).

*M. oryzae* strain expressing Abp1 (MGG_06358.6)-RFP was generated [49] by ATMT method. *M. oryzae* strain expressing RFP-Tef2 was generated as follows: the Tef2 promoter (1 Kb immediately upstream of ATG) and first 1 Kb of Tef2 orf were cloned at *BamH*I/*Spe*I and *Mfe*I/*Hind*III sites in pFGL557, respectively. The resultant final construct pFGL872 was introduced in to wild type *M. oryzae* by ATMT method.

### Isolation of ATS from *abc3*Δ strain of *M. oryzae*

Conidia were harvested from 8- to 9-day old *Magnaporthe* cultures (wild-type Guy11, or the isogenic *abc3*Δ strain) and suspended in de-ionized water to get a count of approximately 1×10^6^ conidia per ml. Two hundred microlitres each of such conidial suspension was placed on to glass coverslips or square sheets (100 Deckglaser, Thermo Scientific) and the conidia were allowed to germinate and form mature appressoria in 24 h under high humidity. Upon incubation, the liquid surrounding the appressoria was collected and saved as total extracellular fluid. The appressoria on each coverslip were covered with 100 µl of hypertonic solution (0.5 M NaCl) and incubated for further 5 h under humid conditions in dark. Appressorial content released into the hypertonic solution was collected and saved as “appressorial extract”. A cell scraper (Corning Incorporated, USA) was used to collect the appressorial debris attached to the coverslips and mixed with the above appressorial extract and saved as total appressorial extract, which was first lyophilized and then extracted with methanol∶chloroform (1∶1) mixture. The resultant extract was again lyophilised, resuspended in de-ionised water, and filtered through a 0.2 µm sterile nylon membrane. The resultant filtered extract was size-fractionated and desalted using a ‘Hi-Trap’ column on an analytical-scale (bed volume, 5 ml; GE Healthcare Life Sciences, Sweden) as per the manufacturer's instructions. Elution was performed with sterile de-ionized water with the flow rate of 1 ml/min and as 0.5 ml fractions. Fractions of interest (displaying cytotoxicity in the yeast cell based assay described in the main Materials and Methods section) were pooled and re-loaded onto the same ‘Hi-Trap’ desalting column for further separation using the elution conditions mentioned above. The instrument used for this chromatographic elution was that for Fast Performance Liquid Chromatography (FPLC) (Amersham, GE Healthcare, Sweden). Fraction(s) from the second round of size based separation on desalting column were then loaded onto an analytical grade C18 reverse phase HPLC column (Phenomenex, USA) and adsorbed materials eluted with a solvent containing 30% acetonitrile and 0.1% formic acid. The elution was carried out under isocratic conditions with 0.5 ml/min flow rate and 0.5 ml fraction volume. The fractions collected from FPLC were again tested in the aforementioned yeast cell based assay. Usually, at this stage, a single fraction containing a single peak displayed the characteristic cytotoxic activity and was therefore subsequently used as purified ATS.

### Structural analysis of ATS

Purified ATS was run on an Agilent high performance liquid chromatography (HPLC) 1200 system (Agilent Technologies) before introduction into a 3200 Q-Trap mass spectrometer with a mass accuracy of 20 mmu (Applied Biosystems). HPLC conditions used were as follows: column, Eclipse XDB-C18 (5 µm, 4.6×150 mm, Agilent Technologies, USA); mobile phase, methanol∶water (3∶1) with a flow rate of 0.4 ml/min. The mass spectrometer was operated under atmospheric pressure chemical ionization (APCI) mode, while collision energy (CE) and collision energy spread (CES) of 40 V and 15 V, respectively, were used for tandem mass spectrometry.

### Enzyme Linked Immunosorbent assays for digoxin or ATS

ELISA tests were performed using a set of different concentrations of digoxin (Sigma Aldrich, USA) and monoclonal anti-digoxin antibodies (Sigma Aldrich, USA). Purified ATS (50 µl) or standard digoxin (6 ng to 6 µg) was coated onto ELISA plate. The wells were later blocked overnight at 4°C with 10% calf serum in 1×PBS containing 0.05% Tween20. Monoclonal antibodies (1∶5000) against digoxin used as primary antiserum were added to the wells and incubated for 2 h. After incubation, the wells were washed 4 times for 15 min each with blocking buffer used above, followed by incubation with HRP conjugated anti-mouse IgG (2°) antibodies. Wells were washed in a similar way with 1×PBS containing 0.05% Tween20 after incubation with secondary antiserum for 1 h. Ready to use TMB substrate (Sigma Aldrich, USA) was added to the wells to test HRP activity. Assays either without antigen (digoxin or ATS) or without primary antiserum were run in parallel as negative controls.

### Growth inhibition assays

Approximately, 3 µl of 1×10^7^ cells/ml from overnight grown wild-type *S. cerevisiae*, wild-type *S. pombe* (MBY104) or MBY104 expressing the *M. oryzae ABC3* (MBY2838, Supplementary Table 2 online) were inoculated in 150 µl YES medium in a 96-well plate. The cells were incubated with constant shaking at 25°C in the presence of 50 µl of de-ionised water or residual solvent prepared from any other FPLC fraction (untreated or solvent control, respectively) or purified fraction (treated). Cell density (OD at 600 nm) of untreated or treated wild-type yeast cells was checked every hour over a 10 h period. To study cell wall biogenesis in control and treated samples, the cells were harvested, washed, stained with calcofluor white after 6 h of incubation, and examined using an epifluorescence microscope (Olympus IX71, Japan). To estimate Minimum Inhibitory Concentration (MIC), approximately, 1×10^7^ cells/ml from an overnight grown culture of MBY104 were inoculated in 20 ml fresh YES medium in 250 ml flasks. The cells were incubated at 25°C on a shaker in the absence or presence of different concentrations of digoxin (Sigma Aldrich, USA). A stock of 1 mM digoxin, digoxigenin, or ouabain (Sigma Aldrich, USA) was prepared by adding 7.8 mg, 3.9 mg, and 7.3 mg, respectively, in 10 ml of 50% ethanol. A working stock of 200 µM solution was prepared by diluting 1 mM stock with fresh YES medium. Further dilutions were made from this working stock by adjusting total volume with fresh YES to 20 ml. Cell density of untreated (YES containing 5% ethanol) or treated *S. pombe* cells was checked in terms of absorbance after every one hour over 3–4 generations. Experiments were performed in duplicate and confirmed by several biological replicates. Wild-type *C. albicans* strain SC5314 (a kind gift from Wang Yue, Singapore) was grown in YPD broth overnight at room temperature. Approximately 3 µl of 1×10^7^ cells/ml culture was inoculated in 150 µl of fresh YPD medium dispensed in a 96-well plate. The yeast cells were treated in a similar way as *S. pombe* above. For induction of hyphal growth in *Candida* strain, 10% calf serum was added to the YPD medium and the cells were grown at 37°C for 6 h. For microscopic observation (both yeast as well as hyphae), the cells were stained with calcofluor white after 6 h of incubation with or without ATS or digoxin.

To study the effect of ATS on Guy11, 1 µl of a conidial suspension (ca. 1×10^6^ conidia/ml) was mixed with 20 µl of water or purified ATS (∼5 ng) and drop-inoculated onto 0.6% agarose gel and incubated for 4 h. Cells were stained with calcofluor white, washed and observed using an epifluorescence microscope.

### Immunoprecipitation assay

Mycelia used for total protein extraction was obtained by growing the relevant strains in liquid CM with gentle shaking for 2–3 days. For total protein extractions, CM-grown mycelia were ground to a fine powder in liquid nitrogen and re-suspended in 0.5 ml of 1×PBS. Lysates were cleared by centrifugation at 12000 g for 10 min at 4°C. The lysate was then divided in to 2 equal parts – one was mixed with ATS and incubated on ice for 2 h whereas the other part was used as a control. Monoclonal anti-digoxin Ab was then added to these 2 samples and incubated at 4°C for 1 h. The pull down assay was performed using Protein A Sepharose 4 Fast Flow (GE Healthcare Biosciences, USA). To identify the pulled-down proteins, the final IP sample was fractionated by SDS-PAGE, and silver-stained using a kit (Silver Stain Plus kit, BioRad). The protein of interest was digested using a Trypsin In-gel Digestion Procedure (http://www.proteomecenter.org/ under Protocols) and processed for mass spectrometric analysis. MS Instrument used for MALDI-Tof-Tof MS was 4700 Proteomics Analyzer (Applied Biosystems). Database searching for protein matches was performed using Data Explorer v4.6 (Applied Biosystems) by comparing peptide masses with those in the NCBI protein database. The searches were conducted with the following criteria: S/N Ratio in MS/MS mode for peak identification >40; with carbamidomethylation of cysteine (fixed modification) and methionine oxidation (variable modification); using NCBInr Protein Database, selecting all entries, using the parent ion mass with an error tolerance of 100 ppm and MS/MS fragment mass tolerance of 0.2 Da.

### Recording of cardiac activity in zebrafish larvae

Wild-type zebrafish (*Danio rerio*, TU) were reared under standard laboratory conditions at 28°C. A working concentration of 415 nM ATS was prepared in fish water. Embryos (n = 5 each/experiment) at 0 to 1 hour post fertilization (hpf) were incubated in either ATS- (100 ng/300 µl) containing fish water or the solvent control (prepared from any other FPLC fraction collected during ATS purification) and observed over 3 dpf. Bright field images and videos (streaming with time lapse 40 ms per frame, 150 frames over 5.7 sec) were taken using Zeiss Axioplan 2 microscope equipped with a CCD camera. The heart rates (in terms of beats/min) of control and ATS- or digoxin-treated larvae were estimated using a digital chronometer.

### Visualization of the F-actin cytoskeleton

*S. pombe* strain expressing either GFP-CHD (calponin homology domain of the Rng2 protein) or Lifeact-GFP [50] were used to visualize the F-actin cytoskeleton. A 6 h treatment was used to study the effect of ATS, digoxin or calcium (150 mM) on requisite *S. pombe* strains. The cells were observed using a spinning disk confocal microscope and the images processed with MetaMorph software. F-actin dynamics were recorded by taking time-lapse images (z-stack sections covering 4.5 µm with a 0.5 micron step) with an interval of 2 min over 2 h. In parallel, *S. pombe* cells were fixed using paraformaldehyde and stained with Alexa Fluor 488 Phalloidin (Life Technologies, USA), and observed using a Zeiss LSM 510 inverted confocal microscope. *M. oryzae* strain expressing Abp1-RFP or RFP-Tef2 was observed using a Yokogawa spinning disk inverted confocal or Olympus BX51, respectively. F-actin dynamics were recorded by taking time-lapse images (z-stack sections covering 3 µm with a 0.5 micron step) with an interval of 15 sec over 5 min.

### Real Time qRT-PCR

Detached rice (CO39) leaves were drop-inoculated with residual solvent or ATS (∼5 ng per drop) and incubated for 24 h. Total RNA was extracted from these leaf tissues as per the manufacturer's instructions (RNeasy Plant Mini kit, QIAGEN, USA). qRT-PCR was performed on ABI 7900HT (Applied Biosystems, USA) using SYBR Green I and the requisite primer sets (Table S1 in Text S1) for *Oryza sativa*-specific open reading frames including PR1a, PR5, peroxidase, and tubulin.

### Surface inoculation assays on leaf explants

A 20 µl drop of residual solvent control or purified ATS (∼5 ng) was inoculated onto detached rice (CO39) or barley leaf blade and incubated for 48 to 72 h. Barley leaf blades incubated for 72 h were tested for cell viability by staining with Trypan Blue (Sigma Aldrich, USA). Similarly, rice (CO39) leaf blades incubated with ATS, digoxin, or residual solvent control for 48 h were examined for H_2_O_2_ production by taking ultrathin sections of the inoculated area, followed by staining with cerium chloride (CeCl3) as described [51].

### Host penetration assays

Approximately 1000 conidia per 10 µl droplet from the wild-type strain (Guy11) were inoculated to test the penetration of rice leaf sheath cells. To test the effect of ATS on penetration ability, 10^3^ wild-type conidia resuspended in 10 µl of either sterile plain (control) or ∼5 ng ATS-containing de-ionised water were inoculated onto rice leaf sheath for 24–30 h under humid conditions. To test the effect of Ca^+2^ on penetration efficiency, required concentration of CaCl_2_ was added to the inoculated area at either 7 or 23 hpi in a separate experiment. *M. oryzae* invasion of the host tissue was quantified through aniline blue-stained penetration pegs (papillary callose deposits by the host) underneath the appressoria or by directly observing penetration hyphae using DIC optics. Aniline blue-stained callose papillae were observed by epifluorescent illumination (360 nm excitation) on an Olympus IX71 microscope.

### Blast disease inhibition assay

Approximately 200 conidia (in 20 µl H_2_O) from the wild-type Guy11 strain were inoculated on barley (or rice) leaf explants to study the disease reaction in the presence or absence of digoxin (200 µM). Blast disease symptoms or HR reaction (if any) were scored by direct and/or microscopic observation at 6 dpi.

### Statistical analyses

Statistical data involving analysis of transcript levels of PR genes or appressorial function in the presence of excess Ca^+2^ or ATS were evaluated by one-way ANOVA (analysis of variance) or the Student's T-test.

## Supporting Information

Text S1 **Supporting figures and tables.** Details about (a) purification, ELISA assay, and estimation of ATS (b) testing and MIC of ATS and related steroidal glycosides (Digoxin, Digoxigenin, Ouabain) on yeast (c) Relationship between ATS, Tef2 and the F-actin cytoskeleton. (d) Effect of ATS on zebrafish heart function (e) Yeast strains and oligonucleotide primers used in this study.
(DOC)

Video S1 **Heart rate in zebrafish larvae treated with residual solvent.**
(MOV)

Video S2 **Heart rate in ATS-treated zebrafish larvae.**
(MOV)

Video S3 **Morphology and dynamics of the GFP-labelled F-actin cytoskeleton in** ***S. pombe*** **treated with residual solvent.**
(MOV)

Video S4 **Effect of ATS on the F-actin cytoskeleton in wild-type** ***S. pombe*** **.**
(MOV)

Video S5 **F-actin cytoskeleton in wild-type** ***S. pombe*** **treated with digoxin.**
(MOV)

Video S6 **F-actin cytoskeleton in the** ***tef2*** **Δ** ***S. pombe*** **cells.**
(MOV)

Video S7 **Effect of excess Ca^+2^ on the F-actin cytoskeleton in wild-type** ***S. pombe*** **.**
(MOV)
