# Supplementary material for: Abc3-Mediated Efflux of an Endogenous Digoxin-like Steroidal Glycoside by Magnaporthe oryzae Is Necessary for Host Invasion during Blast Disease
Source: PLoS Pathog. 2012 Aug 23;8(8):e1002888. doi: 10.1371/journal.ppat.1002888 (PMC3426555; doi:10.1371/journal.ppat.1002888)
Supplement: Text S1 — Supporting figures and tables. Details about (a) purification, ELISA assay, and estimation of ATS (b) testing and MIC of ATS and related steroidal glycosides (Digoxin, Digoxigenin, Ouabain) on yeast (c) Relationship between ATS, Tef2 and the F-actin cytoskeleton. (d) Effect of ATS on zebrafish heart function (e) Yeast strains and oligonucleotide primers used in this study. (DOC) [file ppat.1002888.s001.doc]

**Figure S1:**

**Estimation of concentration of purified ATS by ELISA using monoclonal anti-digoxin antibodies.** **(A)** Colorimetric detection of reactivity towards monoclonal anti-digoxin antibodies. (-) 1° Ab, monoclonal anti-digoxin antibody control; (-) Ag, digoxin control. **(B)** Graphical representation of the standard plot for digoxin using indicated concentration and corresponding absorbance at 450 nm. **(C)** ELISA-based estimation of ATS present in the appressorial extract (A/E) or extracellular fluid (E/F) from the wild-type or *abc3*Δ. Asterisk indicates that the colorimetric detection of the reactivity towards anti-digoxin antibodies was beyond sensitivity range.

**A**

**B**

**C**

**Figure S2:**

**ATS and digoxin share a characteristic dose-dependent inhibitory activity.** Growth curves of wild-type **(A, C, and D)** or Abc3-expressing **(B and D)** *S. pombe* cells grown in the presence of the indicated concentrations of ATS **(A and B)** or digoxin **(C and D)**. Thick and thin lines in same color in (**D)** denote comparative growth curves of wild type and Abc3-expressing strain, respectively, in the presence of a given concentration of digoxin. Data represent mean ± SE of two biological replicates. **(E)** Wild-type *S. cerevisiae*, or *C. albicans* cells were treated with residual solvent, ATS, or digoxin for 6 h and stained with CFW. Arrowheads show aberrant septal/cell wall biogenesis. Bars = 5 μm.

**E**

**Figure S3:**

**MIC of digoxigenin (A) and ouabain (B) against wild-type *S. pombe*.** The bar charts represent results from two replicates each.

**Figure S4:**

**Exogenous ATS slows down the heart rate in zebrafish larvae.** Zebrafish embryos at 1 hpf were treated with 415 nM ATS, digoxin, or residual solvent, and observed under bright field microscope to monitor the heart development and function over 3 days post fertilization. Bar chart shows the heart rates (beats/min) of the larvae treated for 26 hpf with the compounds mentioned. The data represent mean ± SEM from three independent experiments.

**Figure S5:**

**Tef2 associates with ATS in *M. oryzae*. (A)** Co-immunoprecipitation assay using total protein extract from the wild-type *M. oryzae*, ATS, and monoclonal anti-digoxin antibodies. Arrowhead indicates Tef2 as one of the polypeptides bound to ATS, and which was identified later by constituent peptide fragments (in red; *P* < 0.05) through mass spectrometric analysis shown in **(B)**.

**A**

**B**

**MGNKEKSHLN VVVIGHVDSG KSTTTGHLIY KLKGIDQRTI EKYEKEAAEL GKGSFKYAWV LDKLKAERER GITIDIALWK FETAKYQVTV IDAPGHRDFI KNMITGTSQA DCAILVIGAG TGEFEAGISK DGQTREHALL AFTLGVRQLI VAVNKMDTAK WAQSRYDEIV KETSNFLKKI GFNPDSVPFV PISGFNGDHM**

**ISESADIKGN ISPNAPWYKG WTKTVNKDGK KEKVIGGASL QDAIDDVTPP TRPTDKPLRL PLQDVYKIGG IGTVPVGRIE TGILKPGMVV TFAPANVTTE VKSVEMHHQQ LPEGVPGDNV GFNVKNVSVK DIRRGNVAGD SKNDPPMGCA SFNAQVIILN HPGQVGAGYA PVLDCHTAHI ACKFSEILEK LDRRTGKSIE**

**SNPKFIKSGD AAIVKMIPSK PMCVETFSEY PPLGRFAVRD**

**MRQTVAVGVI KSVDKSQGTQ GKVTKSAAKA AKK**­­­­

**Figure S6:**

**F-actin cytoskeleton in *S. pombe* treated with ATS or digoxin.** The *tef2*Δ mutant (untreated) or wild-type cells treated with residual solvent (control), ATS, digoxin, or Ca^+2^ for 6 hours were fixed and stained with Alexa Fluor 488 Phalloidin. Arrowheads show aberrant accumulation of F-actin patches and cables at the cell end(s). Bars = 10 μm.

**Table S1: List of oligonucleotide primers used in this study**.

| **Name** | **Sequence** |
| --- | --- |
| PR1aF (GenBank: EF061246.1) | AAGTACGGCGAGAACATCT |
| PR1aR | GTCGTACCACTGCTTCTCC |
| PR5F (GenBank: AC137991.3) | GGTGTTCAAGCAGGACCAGT |
| PR5R | CCCTTGAAGAACTGCGAGTA |
| PeroxF (GenBank: X66125.1) | CTTAGCTTAATGCTGCTGGT |
| PeroxR | GCACGACGTGTCATAGAAC |
| TubF (GenBank: AF030548.1) | ACTGATGTCGCTGTTCTTCT |
| TubR | CTGTTGAGGTTGGTGTAGGT |
| Tef2FsalI (GenBank: NM_001022750.1) | GAGAGTGTTgtcgacATGGGCAAGGAAAAGGGAC |
| Tef2RbamHI | GAGAGTGAggatccCTTCTTGGCGCCAGCCTTAAC |
| MoTef2MFTPromF | GAGAGTGTTggatccGACTTGCTGACACCCTGTTC |
| MoTef2MFTPromR | GAGAGTGTTactagtTTTGGCGGTTTGGTGCTCTC |
| MoTef2MFTOrfF (GenBank: XP_361098.1) | GAGAGTGTTcaattgATGGGTAACAAGGAGAAG |
| MoTef2MFTOrfR | GAGAGTGTTaagcttTAATCATGTTCTTGATG |

Note: Lower case letters in the primer sequences denote Restriction Enzyme sites.

**Table S2:** List of fission yeast strains used in this study.

| **Strain** | **Genotype** | **Reference** |
| --- | --- | --- |
| MBY104 | *ade6-21 ura4-D18 leu1-32 h^+^* |  |
| MBY2838 | *Moabc3^+^-leu1^+^ade6-M216 leu1-32 ura4-D18 his3-D1 h^-^* | [This](#_ENREF_2) study |
| MBY2440 | *swo1-GFP-ura4^+^ ura4-D18 leu1-32 h^-^* |  |
| MBY2309 | *nmt41-GFP-CHD-leu1^+^ leu1-32 ade6-216 ura4-D18 h^-^* |  |
| *tef2*Δ | *tef2Δ::ura4^+^ade6-216 ura4-D18 leu1-32 h^+^* | This study |
| Tef2-RFP | *tef2-RFP-ura4^+^ade6-216 ura4-D18 leu1-32 h^+^* | This study |

**References specific to Table S2**

1. Wong KC, Naqvi NI, Iino Y, Yamamoto M, Balasubramanian MK (2000) Fission yeast Rng3p: an UCS-domain protein that mediates myosin II assembly during cytokinesis. J Cell Sci 113 ( Pt 13): 2421-2432.

2. Mishra M, D'souza VM, Chang KC, Huang YY, Balasubramanian MK (2005) Hsp90 protein in fission yeast swo1p and UCS protein Rng3p facilitate myosin II assembly and function. Eukaryot Cell 4: 567-576.

3. Karagiannis J, Bimbo A, Rajagopalan S, Liu J, Balasubramanian MK (2005) The nuclear kinase Lsk1p positively regulates the septation initiation network and promotes the successful completion of cytokinesis in response to perturbation of the actomyosin ring in Schizosaccharomyces pombe. Mol Biol Cell 16: 358-371.
